# Relationships between lymphocyte counts and treatment-related toxicities and clinical responses in patients with solid tumors treated with PD-1 checkpoint inhibitors

**DOI:** 10.18632/oncotarget.23217

**Published:** 2017-12-14

**Authors:** Adam Diehl, Mark Yarchoan, Alex Hopkins, Elizabeth Jaffee, Stuart A. Grossman

**Affiliations:** ^1^ Department of Medicine at The Johns Hopkins Hospital, Baltimore, MD, USA; ^2^ The Sidney Kimmel Comprehensive Cancer Center at Johns Hopkins, Baltimore, MD, USA

**Keywords:** lymphopenia, PD-1 inhibitor, response, immune-related adverse event, radiation

## Abstract

The relationships between absolute lymphocyte counts (ALC), drug- related toxicities, and clinical responses remain unclear in cancer patients treated with PD-1 (programmed cell death 1) inhibitors. We performed a retrospective review of 167 adult solid tumor patients treated with nivolumab or pembrolizumab at a single institution between January 2015 and November 2016. Patients with an ALC >2000 at baseline had an increased risk of irAE (OR 1.996, p<0.05) on multivariate analysis. In a multivariate proportional hazards model, a shorter time to progression was noted in patients who were lymphopenic at baseline (HR 1.45 (p<0.05)) and at three months (HR 2.01 (p<0.05)). Patients with baseline lymphopenia and persistent lymphopenia at month 3 had a shorter time to progression compared to those who had baseline lymphopenia but recovered with ALC > 1000 at 3 months (HR 2.76, p<0.05). Prior radiation therapy was the characteristic most strongly associated with lymphopenia at 3 months (OR 2.24, p<0.001). These data suggest that patients with higher baseline lymphocyte counts have a greater risk for irAE, whereas patients with lymphopenia at baseline and persistent lymphopenia while on therapy have a shorter time to progression on these agents. These associations require further validation in additional patient cohorts.

## INTRODUCTION

Programmed cell death protein 1 (PD-1) is a molecule that modulates cellular immunity to limit autoimmunity, but can also be co-opted by cancers and infections to create immune tolerance [1]. Nivolumab and pembrolizumab are fully human IgG4 programmed death 1 (PD-1) checkpoint–inhibitor antibodies that selectively block the interaction of the PD-1 receptor with its two known ligands, programmed death ligand 1 and 2 (PD-L1 and PD-L2). By blocking the interaction of PD-1 with its ligands, these therapies halt the negative signal that downregulates T-cell activation [2]. Nivolumab and pembrolizumab have significant clinical activity in multiple tumor types, including squamous and non-squamous non–small-cell lung cancer, melanoma, renal cell carcinoma (RCC), urothelial carcinoma, and head and neck squamous cell carcinoma (HNSCC) [3–11]. Overall response rates have been up to 30 - 40% for melanoma, up to 20% for NSCLC, and up to 25% in RCC treated with PD-1 inhibitor monotherapy; however, the most remarkable aspect of this novel drug class is the durability of responses observed in a subgroup of responders [11].

Inhibition of the PD-1 checkpoint can result in immune activation in non-target tissues, resulting in immune-related adverse events (irAE) in a subset of patients. The risk of irAEs is higher in patients receiving PD-1 inhibitor therapy in combination with other immune checkpoint therapies such as ipilimumab, an inhibitor of cytotoxic T-lymphocyte-associated protein 4 (CTLA-4). For patients receiving combination therapy with a PD-1 and CTLA-4 inhibitor, the rate of grade 3 or 4 adverse events is as high as 55%[12].

The discovery of factors that influence the clinical response to immunotherapy remains an area of active research and is important to maximizing the benefit/risk ratio of these agents in clinical practice. Moreover, factors that serve as a marker of anti-tumor effect can aid in the discovery of new immunotherapy combinations that augment sub-optimal responses to monotherapy. In this single center retrospective cohort study of patients receiving PD-1 inhibitor therapy for solid tumors, we analyzed the relationship between absolute lymphocyte count (ALC) and rates of irAEs and objective responses.

## RESULTS

Of the 167 patients included in our analysis, 54 had lung cancer, 60 had melanoma, 25 had RCC, 12 had urothelial, 8 had HNSCC, 6 had Merkel cell carcinoma, and 2 had MMR-d colon cancer. Patient and treatment characteristics are contained in Table 1. Nivolumab was prescribed to 75% of patients, with all others receiving pembrolizumab. Fifty-one percent had received prior radiation therapy and 75% had received prior chemotherapy. Eleven percent of patients received prior ipilimumab therapy as one of their prior chemotherapy lines, and 17% of patients received concurrent ipilimumab therapy with their PD-1 inhibitor. At database lock, 53% of patients were on therapy with a PD-1 inhibitor. The median duration on therapy with the PD-1 inhibitor was 6.6 months. The median baseline and three-month absolute lymphocyte counts (ALC) were 1310 and 1220, respectively. Lymphopenia (ALC<1000) was present in 29.9% and 31.0% at baseline and 3 months after treatment initiation, respectively. The median follow-up time was 9.6 months with the longest follow-up time of 111 months. In this limited follow-up time, there were 21 deaths in total leading to an overall survival of 87.4%. There were 68 responders (15 CR and 53 PR), yielding an overall response rate of 41%. Ultimately, 74 patients (44%) developed progressive disease with or without an initial response to therapy and the median time to progression was 2.8 months.

**Table 1:** Patient characteristics

	Number	%	% in those with irAE	% in those without irAE	P value	% in those with response	% in those without a response	P value
**Gender**								
Male	99	59.28%	59	60	P = 1.000	54	63	P = 0.337
Female	68	40.71%	41	40		46	37	
**Age**								
<50	18	10.78%	14	10	P = 0.425	12	10	P = 0.802
50 - 75	118	70.66%	67	72	P = 0.465	72	70	P = 0.862
>75	31	18.56%	20	18	P = 0.831	16	20	P = 0.550
**Race**								
White	136	81.43%	92	76	P = 0.0685	88	77	P = 0.181
Black	22	13.17%	4	17		11	15	
Hispanic	4	2.40%	4	2		0	4	
Asian	3	1.80%	0	3		0	3	
Other	2	1.20%	0	2		1	1	
**Tumor Type**								
Lung	54	32.34%	23	36	P = 0.0065	21	41	P = 0.0004
Melanoma	60	35.93%	57	27		53	24	
RCC	25	14.97%	12	16		7	20	
HNSCC	8	4.79%	0	7		4	5	
Urothelial	12	7.19%	6	8		12	4	
Other (Merkel Cell Carcinoma, Colon Cancer)	8	4.79%	2	6		3	6	
**PD1 Inhibitor**								
Pembrolizumab	42	25.00%	25	25	P = 1.000	35	18	P = 0.018
Nivolumab	125	75.00%	75	75		65	82	
**Prior XRT**								
No	82	49.10%	57	46	P = 0.239	54	45	P = 0.273
Yes	85	50.90%	43	54		46	55	
**Prior Chemotherapy**								
No	42	25.10%	43	17	P = 0.0008	29	22	P = 0.364
Yes	125	74.90%	57	83		71	78	
**Prior Ipilimumab**								
No	148	88.62%	82	91	P = 0.113	85	91	P = 0.323
Yes	19	11.38%	18	9		15	9	
**Number of Prior Chemotherapy Regimens**								
1	76	60.80%	73	57	P = 0.012	56	64	P = 0.423
2	26	20.80%	14	23		29	16	
3	14	11.20%	7	13		13	10	
4	6	4.80%	3	5		2	7	
5	1	0.80%	0	1		0	1	
6	1	0.80%	0	1		0	1	
7	1	0.80%	3	0		0	1	
**Concurrent Treatment with Ipilimumab**								
No	139	83.23%	69	90	P = 0.0014	75	89	P = 0.021
Yes	28	16.77%	31	10		25	11	
**Death**								
No	146	87.43%	86	88	P = 0.802	97	81	P = 0.0016
Yes	21	12.57%	14	12		3	19	
**Immune Related Adverse Event**								
No	116	69.46%				63	74	P = 0.173
Yes	51	30.54%				37	26	
**Number of irAE**								
1	39	76.47%				68	85	P = 0.221
2	10	19.61%				24	15	
3	2	3.82%				8	0	
**Immune Related Adverse Event Requiring Treatment**								
No	43	25.75%				66	80	P = 0.071
Yes	124	74.25%				34	20	
**Immune Related Adverse Event Grade**								
1	17	33.33%				63	74	P = 0.526
2	19	37.25%				10	10	
3	13	25.49%				16	8	
4	2	3.92%				9	7	
**Median Treatment Duration (months)**	6.6		6.06	6.68		11.13	4.66	
**Mean Treatment Duration (months)**	9.16		9.18	9.14	P = 0.976	12.9	6.58	P < 0.0001

Table of patient and treatment characteristics including demographics, tumor type, PD-1 inhibitor used, prior treatments, immune-related adverse events and treatment duration with comparisons between those with and without response and with and without irAE. P values greater than 0.05 indicate no significant difference in the characteristic between those with and without irAE or those with and without response. The P value was calculated using the appropriate statistical test (2-tailed Fisher’s exact test for binary data, Pearson’s chi-squared test for sets of categorical data, *t* test for continuous dependent variable).

### Patient characteristics associated with lymphopenia

Table 1 contains percentages of patients with various demographic and treatment characteristics including stratification by response to therapy as well as occurrence of irAE. In univariate analysis, the frequency of lymphopenia (ALC<1000) at baseline was no different in those who had received prior radiation and those who had not. However, at 3 months after the start of therapy, the frequency of lymphopenia was significantly higher in those who received prior radiation therapy (p=0.0001). There was no difference in lymphopenia at 3 months between those who had received prior conventional radiation therapy versus prior stereotactic body radiation therapy (SBRT). A similar, but non-significant, trend was seen in those with prior chemotherapy. In univariate analysis, there was no association between prior chemotherapy and baseline lymphopenia. In a multiple logistic regression model including age, sex, ethnicity, tumor type, PD-1 inhibitor used, prior chemotherapy, prior radiation therapy, concurrent ipilimumab and occurrence of irAE, prior radiation therapy was the most significantly associated with lymphopenia at 3 months with OR 2.24 (p<0.001). In this multivariate model, there was no association between prior radiation therapy and lymphopenia at baseline, consistent with the univariate analysis. In addition, there was no association between prior chemotherapy and lymphopenia at baseline or 3 months in the multivariate model. In addition to prior radiation therapy, tumor type was significantly associated with lymphopenia at baseline (p<0.01) and at 3 months (p<0.05) in this multiple logistic regression model, owing to significantly less lymphopenia in those with melanoma relative to other tumor types.

### Relationship between baseline lymphocyte counts and drug-related irAE

A total of 51 patients (30.5%) in this patient population experienced an adverse event of any grade with a median time to develop an irAE of 2.6 months. Categorized by the highest grade irAE experienced, 17 patients (10.1%) experienced Grade 1 irAE, 19 (11.3%) experienced Grade 2 irAE, 13 (7.8%) experienced Grade 3 irAE, and 2 (1.2%) experienced Grade 4 irAE. Of those with an irAE, 43 (84%) required treatment with 32 (63%) requiring systemic steroids and 1 (2%) requiring an immunosuppressive therapy beyond steroids (TNFɑ inhibitor), 18 (35%) required therapy discontinuation due to the irAE, and 5 (9.8%) required hospitalization for their irAE. A list of the various irAE that occurred are shown in Table 2.

**Table 2:** irAE types and grades

Immune related adverse event	Any grade (number of patients)	Any grade (% of all patients)	Grade 3 or 4 (number of patients)	Grade 3 or 4 (% of all patients)
All irAE	51	30.4	15	8.9
**Skin**				
Pruritis	1	0.6	0	0.0
Vitiligo	3	1.8	0	0.0
Rash	19	11.3	2	1.2
**GI**				0.0
Pancreatitis	2	1.2	1	0.6
Enteritis/Colitis	5	3.0	2	1.2
Diarrhea	3	1.8	0	0.0
Hepatitis	6	3.6	3	1.8
**Musculoskeletal**				
Myasthenia Gravis	1	0.6	1	0.6
Arthritis	4	2.4	1	0.6
**Nervous System**				
Sensory neuropathy	1	0.6	0	0.0
**Pulmonary**				
Pneumonitis	9	5.4	2	1.2
**Ophthalmologic**				
Optic Neuritis	1	0.6	1	0.6
**Renal**				
Nephritis	1	0.6	0	0.0
**Heme**				
Thrombocytopenia	1	0.6	1	0.6
**Endocrine**				
Adrenal Insufficiency	1	0.6	0	0.0
Hypothyroidism	4	2.4	0	0.0
Hypophysitis	3	1.8	1	0.6
Sjogren’s disease	1	0.6	0	0.0

Table listing all the various types of irAE that occurred including the number and percentage of high grade irAE.

In univariate analysis, a baseline ALC > 2000 as well as an ALC > 2000 at one month into therapy were associated with increased risk of irAE of grade ≥ 2 (p<0.01). In addition, an ALC > 2000 at one month into therapy was associated with increased risk of all irAE (p<0.05) and irAE requiring treatment (p<0.01). This relationship did not hold for a lower ALC cutoff of 1000. A multiple logistic regression analysis including age, sex, ethnicity, tumor type, PD-1 inhibitor used, number of prior chemotherapies, prior radiation, and concurrent ipilimumab therapy, revealed that an ALC > 2000 at the start of therapy was associated with a higher incidence of irAE of grade ≥ 2 (OR 1.996, p<0.05), as was an ALC > 2000 at 1 month into therapy (OR 1.813, p<0.05). An association between irAE of grade ≥ 2 and higher absolute eosinophil count was also noted. Further details of this multiple logistic regression analysis are provided in Table 3.

**Table 3:** Hazard and odds ratios for multivariate models of progression and irAE occurrence

Cox proportional hazards model variable	Hazard ratio	Upper 95% CI	Lower 95% CI	Wald test, P
ALC < 1000 at baseline	1.445	1.941	1.076	0.0145
ALC < 1000 at 3 months	2.008	2.798	1.441	<0.0001
Difference Between ALC at 3 months and at Baseline	1.001	1.002	1.001	<0.0001
Difference Between ALC at 3 months and at Baseline for increments of 100	1.116	1.178	1.058	<0.0001
ALC at Baseline	0.999	1.000	0.999	0.0358
ALC at Baseline for increments of 100	0.947	0.996	0.901	0.0334
ALC at 3 months	0.999	0.999	0.998	0.0004
ALC at 3 months for increments of 100	0.882	0.946	0.824	0.0004
ANC/ALC ratio at 3 months	1.223	1.313	1.138	<0.0001
Baseline Lymphopenia with Persistence at 3 months (vs Recovery at 3 months)	2.764	7.553	1.011	0.0476
Baseline Lymphopenia with Persistence at 3 months (vs Never Lymphopenic)	1.496	2.156	1.039	0.0305
Baseline Lymphopenia with Recovery at 3 months (vs Never Lymphopenic)	1.061	1.992	0.566	0.8530
No Baseline Lymphopenia with New Lymphopenia at 3 months (vs Never Lymphopenic)	2.451	4.053	1.483	0.0005
No Baseline Lymphopenia with New Lymphopenia at 3 months (vs Always Lymphopenic)	3.093	7.050	1.355	0.0073

Multiple logistic regression of grade >= 2 irAE model variable	OR	95% CI	95% CI	Likelihood ratio test, P
ALC > 2000 at baseline	1.996	3.490	1.155	0.0136
ALC > 2000 at 1 month	1.813	3.248	1.030	0.039
AEC at baseline	1.003	1.006	1.000	0.027
AEC at baseline for increments of 100	1.340	1.742	1.035	0.027
AEC at 1 month (excluding those who received steroids within 1 month)	1.002	1.004	1.000	0.0268
AEC at 1 month (excluding those who received steroids within 1 month) for increments of 100	1.208	1.511	1.020	0.0268

Table of hazard ratios (HR) with corresponding confidence intervals (CI) and p values derived from a Cox proportional hazards model of progression, respectively, as well as OR, CI and p values derived from a multivariate logistic regression model of grade ≥ 2 irAE for the listed variables, adjusted for age, sex, ethnicity, tumor type, PD-1 inhibitor used, number of prior chemotherapies, prior radiation, and concurrent ipilimumab therapy.

### Relationship between lymphopenia and tumor progression

**Figure 1:**
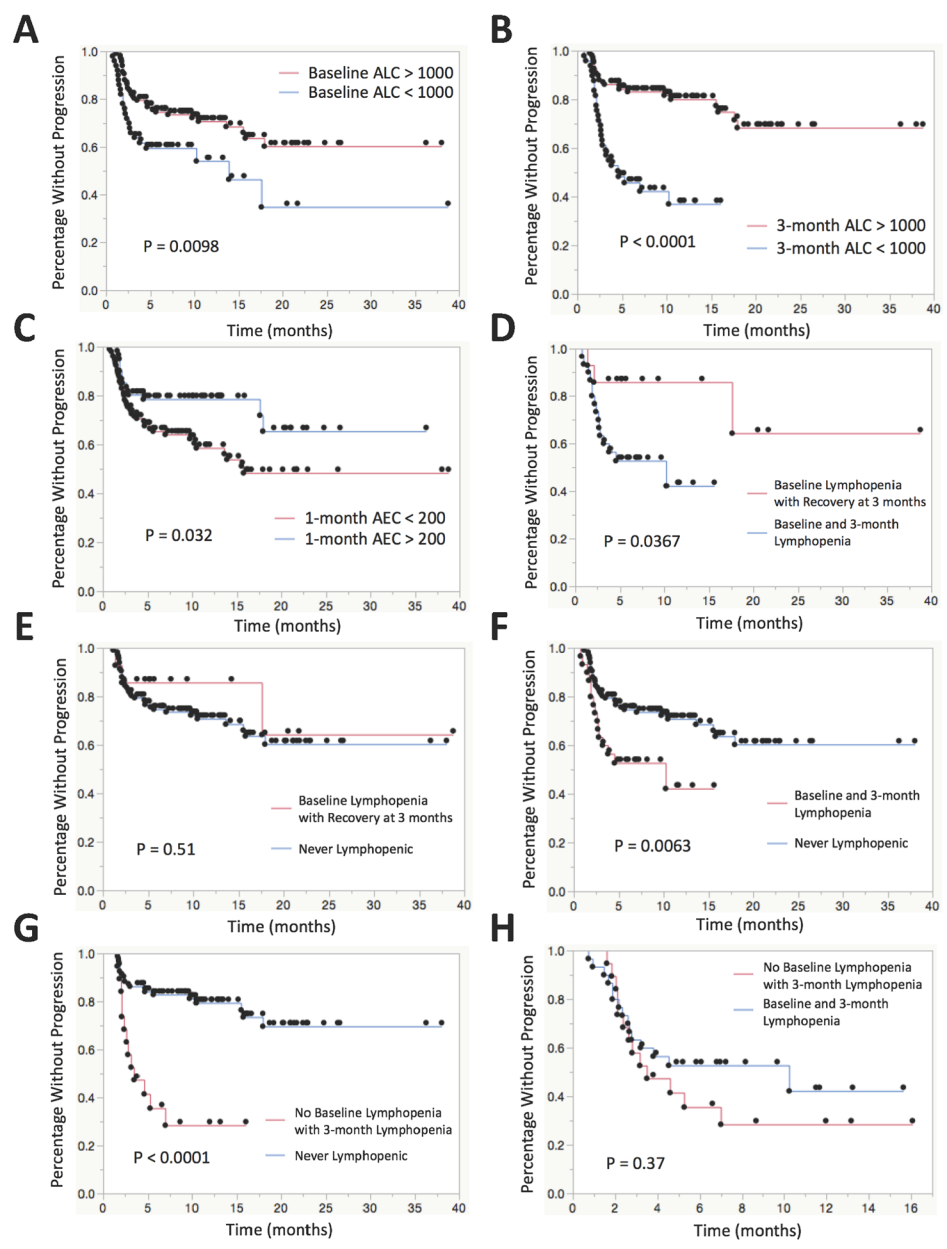
Kaplan-Meier plots for time to progression stratified by various leukocyte subsets **(A)** KM plot comparing patients with baseline lymphopenia (ALC < 1000) vs no baseline lymphopenia. **(B)** KM plot comparing patients with lymphopenia vs no lymphopenia at 3 months after the start of therapy. **(C)** KM plot comparing patients with AEC > 200 vs AEC < 200 at 1 month after the start of therapy. **(D)** KM plot comparing patients who remain lymphopenic at baseline and 3 months after the start of therapy vs patients with baseline lymphopenia who recover to ALC > 1000 at 3 months after the start of therapy. **(E)** KM plot comparing patients with baseline lymphopenia who recover to ALC > 1000 at 3 months after the start of therapy vs patients that are never lymphopenic at baseline or at 3 months. **(F)** KM plot comparing patients who remain lymphopenic at baseline and 3 months after the start of therapy vs patients that are never lymphopenic at baseline or at 3 months. **(G)** KM plot comparing patients who have no baseline lymphopenia who subsequently develop lymphopenia at 3 months after the start of therapy vs patients that are never lymphopenic at baseline or at 3 months. **(H)** KM plot comparing patients who have no baseline lymphopenia who subsequently develop lymphopenia at 3 months after the start of therapy vs patients who remain lymphopenic at baseline and 3 months after the start of therapy.

In univariate survival analysis, the median time to progression was significantly shorter in patients with baseline lymphopenia (13.9 months versus median not reached, p<0.01). Similarly, patients with lymphopenia at 3 months after initiation of treatment progressed more rapidly than other patients (4.6 months vs median not reached, p<0.0001). In patients who were lymphopenic at baseline and had persistent lymphopenia at month 3, median time to progression was 10.2 months, which was significantly shorter than those who had no baseline lymphopenia (median not reached) (p<0.01). However, progression free survival was longer in patients who had baseline lymphopenia but recovered their ALC to greater than 1000 at 3 months (median not reached) (p<0.05). There was no significant difference in time to progression between those with no lymphopenia and those with baseline lymphopenia who recovered with ALC > 1000 at 3 months after the start of therapy (median not reached for either) (p=0.51). Patients who were not lymphopenic at baseline but who became lymphopenic at 3 months had a median time to progression of 3.5 months while those with persistently normal lymphocyte counts fared significantly better (median not reached) (p<0.0001). There was also an association found with absolute eosinophil count > 200 at 1 month as shown in Figure 1 and Table 4.

**Table 4:** Survival analysis by leukocyte subgroups

Categories	Number of patients	Percentage of patients	Median time to progression	Log rank, P	% Without progression at 12 months	SE	Low 95% CI	High 95% CI
Eosinophils at 1 month > 200	61	36.5	Not reached	P=0.032	78.5	5.3	68.1	88.9
Eosinophils at 1 month < 200	106	63.5	15.8		58.6	5.3	48.3	68.9
ALC > 1000 at baseline	117	70.1	Not reached	P=0.0098	70.8	4.4	62.1	79.5
ALC < 1000 at baseline	50	29.9	13.9		54.1	8.2	38.0	70.2
ALC > 1000 at 3 months	109	69.0	Not reached	P<0.0001	80.0	4.1	72.0	88.0
ALC < 1000 at 3 months	49	31.0	4.6		37.0	8.2	20.8	53.1
Baseline lymphopenia with persistence at 3 months	30	20.4	10.2	P=0.0063	42.2	12.0	18.7	65.7
No baseline lymphopenia	117	79.6	Not reached		70.8	4.5	62.1	79.5
Baseline lymphopenia with persistence at 3 months	30	68.2	10.2	p=0.0367	42.1	12.0	18.6	65.6
Baseline lymphopenia with recovery at 3 months	14	31.8	Not reached		85.7	9.4	67.4	104.0
No baseline lymphopenia or lymphopenia at 3 month	95	83.3	Not reached	p<0.0001	79.4	4.5	70.6	88.2
No baseline lymphopenia with subsequent lymphopenia at 3 month	19	16.7	3.5		28.4	11.0	6.8	50.0
Baseline lymphopenia with recovery at 3 months	14	10.7	Not reached	p=0.51	85.7	9.4	67.4	104.0
No baseline lymphopenia	117	89.3	Not reached		70.8	4.5	62.0	79.6
Baseline lymphopenia with persistence at 3 months	30	61.2	10.2	p=0.37	28.4	11.0	6.8	50.0
No baseline lymphopenia with subsequent lymphopenia at 3 month	19	38.8	3.5		42.2	12.0	18.7	65.6

Table of univariate Kaplan-Meier estimates of median survival as well as 1-year survival rate with 95% confidence interval and p-values derived from the log rank test comparing various leukocyte subgroups.

In a Cox proportional hazards model for progression adjusted for age, sex, ethnicity, tumor type, PD-1 inhibitor use, prior radiation therapy, number of prior chemotherapies, concurrent ipilimumab therapy and occurrence of immune-related adverse events, there were a number of associations with lymphopenia and progression as shown in Table 3. Baseline lymphopenia (ALC < 1000) had a significant increased risk of progression with a hazard ratio 1.45 (p<0.05). Baseline ALC as a continuous variable was also associated with progression with hazard ratio 0.947 for every increase in ALC of 100 (p<0.05). In the same model, lymphopenia at 3 months after the start of therapy had an even more significant increased risk of progression with a hazard ratio 2.01 (p<0.0001). Of those patients with lymphopenia at baseline, 30 patients (68%) had persistent lymphopenia (ALC<1000 at baseline persisting to month 3) whereas 14 patients (32%) had normalized lymphocyte counts by month 3. In those patients who were lymphopenic at baseline and had persistent lymphopenia at month 3, there was increased risk of progression compared to those who had baseline lymphopenia but recovered their ALC to greater than 1000 at 3 months with HR 2.76 (p<0.05) and compared to those who were never lymphopenic with HR 1.50 (p<0.05). There was no significant difference in risk of progression between those who were never lymphopenic and those who had baseline lymphopenia but recovered their ALC (p=0.85). In those patients with no lymphopenia at baseline with new lymphopenia at 3 months, there was increased risk of progression compared to those who were never lymphopenic with HR 2.45 (p<0.01) and, interestingly, compared to those who were always lymphopenic at baseline and 3 months with HR 3.09 (p<0.01). We also found associations between progression and ALC at 3 months as a continuous variable, the difference in ALC between baseline and month 3 after the start of therapy, and the neutrophil to lymphocyte ratio at 3 months as shown in Table 3.

## DISCUSSION

This retrospective single institution analysis was designed to investigate the relationships between absolute lymphocyte counts and the toxicity and efficacy of PD-1 inhibitors in patients with solid tumors. Lymphopenia is common in patients with advanced cancers, occurring in approximately 40% of patients receiving radiation therapy for glioblastoma, head and neck cancer, pancreatic cancer, and non-small cell lung cancer [15]. This lymphopenia is profound, with 40% of patients having a CD4 count of <200 cells/mm3, and long-lasting, with low counts commonly persisting for over one year [16].

Our retrospective data suggest that patients with baseline lymphopenia before starting PD-1 inhibitors and those with lymphopenia 3 months after starting therapy may be less likely to benefit from treatment with PD-1 inhibitors, but are also less likely to experience irAEs. Our findings build upon several cohort studies that indicate that peripheral leukocyte populations may be correlated with clinical responses to checkpoint inhibitors. A number of markers for increased ipilimumab efficacy have been described, including high AEC, high ALC and low neutrophil to lymphocyte ratio [17–28]. Similar efforts have been made to predict response to PD-1 inhibitor therapy using peripheral leukocyte counts. In a retrospective analysis of over 600 patients treated with pembrolizumab for metastatic melanoma, baseline relative eosinophil count ≥1.5% and relative lymphocyte count ≥17.5% were found to be correlated with favorable overall survival [29]. In a separate retrospective study of 173 patients with metastatic melanoma treated with checkpoint inhibitors, the presence of eosinophilia at any point in the course of therapy correlated with longer survival [30]. In another retrospective study of 98 patients with unresectable stage III or IV melanoma treated with nivolumab, absolute lymphocyte count >1000 and absolute neutrophil count < 4000 early in the course of therapy at week 3 and 6 were found to be markers of favorable response [31]. These associations require confirmation in prospective clinical trials of immune checkpoint inhibitors.

Additional research is also needed to understand potential mechanisms through which lymphopenia could affect progression free survival for patients receiving an immune checkpoint inhibitor. One hypothesis is that lymphopenia may reflect a state of T cell dysfunction resulting from immune exhaustion and depletion of antitumor lymphocytes, and that these dysfunctional lymphocytes have a limited ability to exert an anti-tumor effect in the setting of PD-1 inhibitor therapy [32]. If this hypothesis is correct, strategies that rescue the T cell repertoire and induce novel T cells capable of an anti-tumor response, such as adoptive cell therapies and vaccination, may be necessary to improve upon response rates in patients with lymphopenia receiving an immune checkpoint inhibitor [33]. Alternatively, lymphopenia may be a prognostic marker resulting from inflammation or other factors that reflect an advanced disease stage. Lymphopenia has been related to survival in a variety of clinical settings, including patients not receiving immune checkpoint inhibitors [15, 34].

In summary, our data indicate that patients with higher baseline lymphocyte counts may have a greater risk for irAE, whereas patients with lymphopenia at baseline and persistent lymphopenia while on therapy have a shorter time to progression on these agents. This analysis has several limitations. This is a single institution study and is therefore subject to the risks of regional and site-specific influences. In addition, given the retrospective nature of the study, we cannot control for patient selection procedures. Furthermore, known prognostic factors that could affect outcome such as ECOG performance status, burden or site of metastases, and PD-L1 status of the tumors were not analyzed in this study. Prospective validation of our results in patients with solid tumors is needed to confirm and expand upon our findings, and improved understanding of the immunology behind this association may lead to the development of more effective therapies for these patients.

## MATERIALS AND METHODS

We performed an IRB-approved retrospective chart review of adult solid tumor patients treated with nivolumab or pembrolizumab at a single institution from January 2015 until November 2016. Solid tumor types that were included were those with FDA approved indications for PD-1 or PD-L1 inhibitor therapy including squamous and non-squamous non–small-cell lung cancer, melanoma, renal cell carcinoma, urothelial carcinoma, HNSCC, Merkel cell and mismatch repair deficient (MMR-d) colon cancer. Patients were excluded if they were receiving PD-1 inhibitors: (a) for hematologic malignancies, (b) concurrently with investigational immunotherapies, (c) on unreported clinical trials, (d) in cancers for which the activity of immune checkpoint inhibitors remains unclear, or (e) for less than two doses of either nivolumab or pembrolizumab. We chose to include patients who received concurrent ipilimumab or had received ipilimumab in a prior line of therapy. Patients were treated until disease progression or until unacceptable toxicity occurred per the discretion of the individual oncologist. Data were collected on patient demographics and treatment history including prior chemotherapy and radiation treatment, response to therapy, adverse events, and leukocyte counts. Response to PD-1 inhibitor therapy was defined using RECIST 1.1 criteria based on imaging done from the start of PD-1 inhibitor therapy to the date of progressive disease or start of a new systemic treatment [13]. Using the RECIST 1.1 criteria, the best response achieved was recorded for each patient and time to response was defined as the earliest time point at which the partial response or complete response category was first achieved. The interval of imaging studies was at the discretion of the individual oncologist but for most patients was approximately every 3 months. Immune-related adverse events (irAE) were defined as adverse events with a potential immunologic basis. Grading of these events used the National Cancer Institute Common Terminology Criteria for Adverse Events (CTCAE) v.4.0 [14]. Data were collected on time to onset of the irAE and subsequent management including requirement for immunosuppressive therapy, PD-1 inhibitor discontinuation, or hospitalization. Leukocyte counts were retrospectively collected at baseline, and at 1, 3 and 6 months after the start of therapy.

Follow-up time was defined from the date of the first dose of PD-1 inhibitor therapy to the date of last known contact or death. Survival probabilities and median survival with 95% confidence intervals (CI) were estimated according to the Kaplan–Meier method, and compared using log-rank tests. Hazard ratios were calculated using the Cox proportional hazards model with P values based on the Wald test. There were no deaths in our cohort that were not considered secondary to cancer. P values for univariate analyses with logistic regression models as well as multivariate regression models were obtained using the likelihood ratio test. P values for univariate analyses with binary variables were calculated using a 2-tail Fisher’s exact test. For univariate analyses with a continuous dependent variable, the *t* test was used for P value calculation. Throughout the analysis, P values less than 0.05 were considered statistically significant. All statistical analyses were performed using JMP software (version 12; SAS institute, Cary, NC).

## Abbreviations

PD-1: programmed cell death 1
PD-L1: programmed cell death ligand 1
CTLA-4: cytotoxic T-lymphocyte-associated protein 4
irAE: immune related adverse events
ALC: absolute lymphocyte count
AEC: absolute eosinophil count
ANC: absolute neutrophil count
HR: hazard ratio
OR: odds ratio
CI: confidence interval
MMR-d: mismatch repair deficient
HNSCC: head and neck squamous cell carcinoma
RCC: renal cell carcinoma
NSCLC: non-small cell lung cancer
ECOG: Eastern Cooperative Oncology Group
